# Impact of the level of pre-ART CD4+ T cells in blood on the rectal HIV reservoir in long-term treated men (VIRECT study)

**DOI:** 10.1186/1471-2334-14-S2-O17

**Published:** 2014-05-23

**Authors:** Delphine Vergnon-Miszczycha, Alexandre Girard, Anne Depincé, Xavier Roblin, Emilie Del Tedesco, Anne Frésard, Claire Guglielminotti, Claude Lambert, Bruno Pozzetto, Frédéric Lucht, Stéphane Paul, Thomas Bourlet

**Affiliations:** 1 University Hospital of Saint-Etienne, Groupe Immunity of the Mucous Membranous and Pathogen Group (GIMAP), EA 3064, Saint-Etienne, France; 2 University Hospital of Saint-Etienne, Infectious Diseases Department, Saint-Etienne, France; 3 University Hospital of Saint-Etienne, Gastroenterology Department, Saint-Etienne, France; 4 University Hospital of Saint-Etienne, Immunology Unit, Saint-Etienne, France; 5 University Hospital of Saint-Etienne, Virology Unit, Saint-Etienne, France

## 

Gut associated lymphoid tissue (GALT) represents the largest reservoir of HIV-1. The aim of the study is: 1) to establish the viral and cellular characteristics of the HIV-1 rectal reservoir from chronically infected men under antiretroviral therapy (ART) for more than one year and less than five years, and, 2) to correlate the size of the rectal reservoir and the cellular composition of rectal mucosa to the level of CD4+ T cells in blood at the start of ART.

For each patient, whole blood and six rectal biopsies were collected. Total HIV DNA and cell-associated HIV RNA were quantified in PBMCs and in rectal samples by rtPCR assays. The cellular composition of blood and rectal samples in CD4+ and CD8+ T cells, Th17 lymphocytes, regulatory T cells (Treg), CD4+/p24+ and CD8+/ PD-1+ T cells was established by flow cytometry.

Up to now, 12 patients were enrolled: 4 with pre-ART CD4+ T cell count above 350/mm3, 3 between 200 and 350/mm3 and 5 under 200/mm3. The mean HIV DNA level in rectal cells was lower in patients who initiated ART with a blood CD4+ T cell count > 350/mm3, compared to those who started ART with a blood CD4+ T cell count < 200/mm3 (3.49 vs 3.74 log10 copies/106 cells). Moreover, the rate of rectal CD8+ T cells exhibiting an exhausted phenotype (CD8+/ PD-1+ T cells) tended to be negatively correlated to the pre-ART blood CD4+ T cell count (p = 0.06, rho = -0.61) and to the rectal [Th17/Treg] ratio (p = 0.058, rho = -0.66).

These preliminary results suggest that initiating ART with a high blood CD4+ T cells (> 350/mm3) limits the size of the HIV rectal reservoir and preserve local immunity, even after long-term therapy. This could participate to decrease local inflammation and viral replication. The study is still ongoing to increase the size of the effective and to complete these results by detecting viral integrated and 2LTR DNA forms, investigating co-infections agents and studying the inflammatory environment.

